# The Bacteriology of Food Poisoning

**Published:** 1913-10-04

**Authors:** 


### THE BACTERIOLOGY OF FOOD POISONING.

### "Ptomaine Poisoning" and Modern Research.

In the Report to the Local Government Board on Bacterial Food Poisoning and Food Infections Dr. Savage has collected a great deal of interesting information, has drawn some very reasonable deductions, and has made certain useful suggestions regarding prevention. The Report deals entirely with disease produced by the infection of food by bacteria which produce toxic substances either before or after digestion, and to which the symptoms of the disease are due. It takes no account of disease produced by the chemical poisons which may be contained in food, such as lead and arsenic, nor does it deal with the infection of food by bacteria associated with specific infectious diseases, such as, for example, typhoid fever or tuberculosis. In other words, the Report deals largely with diseases which until quite recently have been characterised as "ptomaine poisoning"—diseases which in the light of modern investigation we are coming to believe are produced by a definite bacterial infection of the food before it is eaten.

### Three Groups of Bacteria.

Dr. Savage's Report falls naturally into two sections. The first deals with the bacteriology of food poisoning, and the second with the attitude of public health authorities towards food infection. As regards the bacteriology of food poisoning, Dr. Savage points out that the bacteria concerned in food infections can be included roughly in three groups: (*a*) the Gaertner group of bacilli; (*b*) non-Gaertner aërobic bacilli, such as *B. proteus* and *B. coli*; and (*c*) *B. botulinus*.

(*a*) The Gaertner group of bacilli. It seems probable that in the majority of recorded outbreaks of food poisoning in which fatal cases have occurred one or other of the Gaertner group of bacilli is definitely the infecting organism. The Gaertner group of bacilli is a sub-group of the large coli-typhoid group, the members of the Gaertner group occupying an intermediate position between the chemically active coli group and the chemically inert typhoid group, and consisting of: (1) *B. enteritidis*, which not only includes many of the Gaertner bacilli isolated in cases of food poisoning but also many of the strains of *B. typhi murium*; (2) *B. suipestifer*, with which one must associate some of the *B. typhi murium* strains, probably *B. psittacosis*, and all the food poisoning bacilli of this group which are not *B. enteritidis*; (3) *B. paratyphosus B.*, which is the bacillus associated with most of the cases of paratyphoid fever, and is also found in the intestines of people who are carriers of this disease. The *B. morbificans bovis* may be identical with (1) or (2), but it is difficult to place it accurately. In Germany the *B. suipestifer* and the *B. paratyphosus B.* are supposed to be identical, but certain differences have been found between these two by English investigators, particularly by Bainbridge and O'Brien, who show that the two organisms can be differentiated by a series of absorption tests. Bacilli of the Gaertner group have been found in the human intestine, in the intestines of certain animals, and in meat. The *B. paratyphosus B.* is the only member of the Gaertner group which is found at all frequently in the human intestine, and then only in persons who are the subject of paratyphoid fever or who are carriers, acute or chronic, of that disease. Apart from such cases the Gaertner group is seldom found in the human intestine, although in cases of summer diarrhœa *B. enteritidis* and *B. suipestifer* have been isolated. It is therefore reasonable to suppose that the healthy human intestine is naturally free from bacilli of the Gaertner group. In the animal intestine the condition is similar. Many German observers have found *B. suipestifer* in the intestines of newly slaughtered pigs, and *B. enteritidis* and *B. suipestifer* have been isolated frequently from the intestines of rats and mice which were not apparently ill. The occurrence of *B. suipestifer* and *B. enteritidis* in the intestines of apparently healthy rats and mice is a matter of some moment, since rats and mice are very frequently to be found in or about slaughter-houses, warehouses, and dwelling-houses. There seems to be some considerable doubt as to whether *B. enteritidis* and *B. suipestifer* are to be found in the intestines of animals, such as the pig and cattle, which are really healthy; and, while German opinion is somewhat conflicting on this point, it would appear likely that they are not normal inhabitants of the intestines of healthy animals, but are indicative of some pathological condition in the intestine—that is to say, that they are associated with the occurrence of a more or less definite enteritis.

A non-pathogenic variety of this group, which may be called the *para-Gaertner group*, has been apparently isolated from the intestine of the sheep, but it would appear that the sheep is not, commonly at least, a host of the true Gaertner group. As Dr. Savage points out, such para-Gaertner bacilli form a group, for the most part unnamed, which are non-pathogenic, and are not very uncommon in the healthy human intestine and also in the healthy animal intestine. They are capable of differentiation from the true Gaertner group by a series of fermentation and agglutination tests.

### Feeding Experiments.

The virulence of the bacilli of the Gaertner group seems to be very variable, and feeding experiments with true Gaertner bacilli are not very uniform, sometimes producing severe infection with death, sometimes slight infection with recovery, and frequently no symptoms at all. The bacilli themselves are easily killed by heat, being usually destroyed after thirty minutes' exposure to 60° C. The toxins produced by the bacilli are, however, extraordinarily resistant to heat. The toxins of both *B. enteritidis* and *B. suipestifer* have been found to resist heating to 100° C. for thirty minutes. With regard to the heat resisting properties of bacilli and toxins, certain observations by Délépine and Howarth in connection with the Derby outbreak are interesting. They show that the temperature at the centre of a pie which is perhaps a little under-baked does not exceed 47.2° C., and the temperature of the centre of an over-baked pie might not rise above 86.6° C., while there is a difference of several degrees as a rule between the temperature of various pies cooked at the same time. Similarly, brawn or, as Dr. Savage himself pointed out in 1908, sausages might be infected by bacilli of the Gaertner group and cooked apparently quite thoroughly, but the central temperature might not be sufficiently raised to destroy even the bacilli. It can thus be easily seen that, even in meat which has been infected before cooking, certain portions of an apparently well-cooked pie, piece of brawn, or sausage might contain bacilli of the Gaertner group capable of active reproduction and quite virulent. It is obviously very much more difficult to destroy toxins of the Gaertner group by ordinary cooking methods. The ordinary methods of preservation, such as smoking and pickling in brine, do not readily destroy the bacilli of the Gaertner group, but in solutions which contain 15 per cent. or more of salt a rapid destruction of the bacilli takes place.

### Ice-cream and Cheese Poisoning.

(*b*) Non-Gaertner bacilli, such as *B. proteus* and *B. coli*. While it has been thoroughly established that bacilli of the Gaertner group can produce and have produced cases of acute food poisoning, it is not so easy to establish the fact that bacilli of this group have caused any such outbreaks. Organisms of the type of *B. proteus* have been blamed for several outbreaks of food poisoning, but Dr. Savage points out that from the summary of the outbreaks given it was established in none of them that *B. proteus* was the definite etiological factor in the production of the attack, most of the evidence adduced being simply that *B. proteus* was found in the meat which presumably caused the outbreak. It is, however, quite possible that *B. proteus* may be a factor in the production of enteritis produced by contamination of food. *B. proteus*, like the Gaertner group, has been shown to be capable of producing enteritis in animals even after feeding experiments, and although its virulence is even more variable than that of the bacilli of the Gaertner group, and its toxins are more readily destroyed by heat than the toxins of that group, it certainly has a certain pathogenicity to animals, and further investigation on this point would be necessary before one could definitely exclude the *B. proteus* as a possible factor in the production of an outbreak of food poisoning. *B. coli* seems to have played a part in certain cases of ice-cream and cheese poisoning.

(*c*) *B. botulinus*. This organism, which is an anaërobic bacillus, is concerned in outbreaks which even in Germany are fortunately rare. Sausage and ham are the two commonest sources of botulism. From the anaërobic character of the bacillus and the fact that outbreaks of botulism have never followed on the eating of food in a fresh state, it is clear that botulism is produced by an invasion of food by *B. botulinus* only when suitable anaërobic conditions are provided; for example, when a ham is stored at the bottom of a pickling cask and entirely covered by the pickling solution. Van Ermengem states that the disease has never occurred when the food has been properly cooked, as *B. botulinus* and its toxins are easily destroyed by heating. The *B. botulinus* will not grow in media containing more than 6 per cent. of sodium chloride, and it is, therefore, unlikely that any meat would develop *B. botulinus*, even if stored under anaërobic conditions, if a pickling solution containing 15 per cent. of salt were used.

### Infected Potatoes and Mussels.

Dr. Savage's Report also deals at less length with poisonous results produced by infected potato, mussels, and cheese. There seems nothing specific in poisoning by potato; the symptoms are those of infection of food by ordinary organisms. One interesting point is that it seems likely that potatoes only become infected after they are peeled. Mussel poisoning has some clinical symptoms peculiar to itself, and may be due to the presence of some metallic poison, although this must be rather rare. A toxic substance called *mytilotoxin* has been isolated from poisonous mussels, and it seems likely that such mussels have been infected by bacteria, but we know little of any special bacteria concerned in this condition, although it is probable that the mussels become poisonous through contamination by sewage. Similarly, cheese poisoning, which has certain more or less specific clinical manifestations, seems to be the result of the infection of cheese by some definite organism, and at the present date it would seem likely that, as mentioned above, certain virulent strains of *B. coli* are concerned in this infection, or, possibly, a strain of *B. lactis aerogenes*, but further investigation of this point is necessary before any definite conclusion can be reached.

The whole trend of Dr. Savage's Report is to minimise the likelihood of simple putrefactive organisms producing an outbreak of food poisoning, and this attitude is one with which modern workers will sympathise. Putrefying food protects by its very offensiveness. Dr. Savage, however, lays proper stress upon the somewhat complex changes which occur in the initial stages of meat putrefaction, and urges that it is very desirable that investigations from this aspect of the subject should be carried out on modern chemical and bacteriological lines. He does not attempt to exclude the possibility of the occurrence of isolated cases and limited outbreaks of food poisoning being due to the consumption of meat in a state of early decomposition, but points out truly that such outbreaks are usually very limited, and much less virulent than outbreaks caused by infection by the Gaertner group of bacilli.

